# Circulating CTRP7 Is a Potential Predictor for Metabolic Syndrome

**DOI:** 10.3389/fendo.2021.774309

**Published:** 2021-11-11

**Authors:** Wenjing Hu, Bin Zhan, Qinge Li, Gangyi Yang, Mengliu Yang, Minghong Tan, Shan Geng, Hua Liu, Chen Chen, Dongfang Liu, Ling Li

**Affiliations:** ^1^ Key Laboratory of Diagnostic Medicine (Ministry of Education) and Department of Clinical Biochemistry, College of Laboratory Medicine, Chongqing Medical University, Chongqing, China; ^2^ Department of Endocrinology, The Thirteenth People’s Hospital of Chongqing, Chongqing, China; ^3^ Department of Endocrinology, The Second Affiliated Hospital, Chongqing Medical University, Chongqing, China; ^4^ Department of Pediatrics, University of Mississippi Medical Center, Jackson, MS, United States; ^5^ Endocrinology, School of Biomedical Science (SBMS), Faculty of Medicine, University of Queensland, Brisbane, QLD, Australia

**Keywords:** CTRP7, MetS, insulin resistance, interventional tests, Bioinformatics

## Abstract

**Background:**

Previous animal studies have revealed that CTRP7 is related to energy metabolism. However, little is known regarding the relationship between CTRP7 and metabolic diseases in humans. Hence, this study was designed to explore the association between CTRP7 and MetS through a cross-sectional study and multiple intervention studies.

**Methods:**

A total of 624 individuals were enrolled in this study. The levels of CTRP7 and APN were determined by ELISA kit. HEC, OGTT and lipid infusion were performed in heathy individuals to investigate the association of CTRP7 and glucose, insulin and FFA. Bioinformatics analysis was then undertaken to identify genes and signaling pathways associated with CTRP7. The relationship between CTRP7 with MetS components was also evaluated.

**Results:**

In MetS patients, serum CTRP7 concentrations were significantly higher than in healthy controls, and was positively correlated with WC, BP, FBG, 2h-BG and TG, but negatively correlated with HDL-C and APN. Multivariate logistic regression analysis uncovered that CTRP7 was strongly correlated with the occurrence of MetS. In addition, circulating levels of CTRP7 in patients with two or more MetS components were higher than those with one MetS component. In the intervention studies, OGTTs resulted in a significant reduction in serum CTRP7 concentration. However, the increase in insulin levels caused by EHC and the increase of FFA caused by lipid-infusion led to the significant increase of serum CTRP7 concentration. Meanwhile, bioinformatics analysis revealed that CTRP7 was strongly associated with metabolism-related genes and signal pathways, which further illustrate the association of CTRP7 with whole-body metabolism.

**Conclusions:**

Serum CTRP7 is increased in MetS patients, which may be a biomarker related to metabolic diseases.

**Clinical Trial Registration Number:**

ChiCTR2000032878

## Introduction

Metabolic syndrome (MetS), also known as insulin resistance (IR) syndrome, was first described in 1988. It represents an aggregation of cardiovascular risk factors. At present, IR, obesity, impaired glucose tolerance (IGT), dyslipidemia, hypertension, and chronic low-grade inflammation are all considered characteristics of MetS (1–4).

MetS classification has important clinical significance and application value for screening metabolic diseases related to obesity (5). Because characteristics of MetS include chronic low-grade inflammation and IR, it is important to study the biomarkers for prevention and diagnosis as well as finding new drug targets. Recently, we and others have found that members of the CTRP family, such as c1q/tnf related protein subtype 6 (CTRP6), CTRP5, and CTRP15, are associated with the occurrence of MetS, type 2 diabetes mellitus (T2DM), and obesity (6–12). Therefore, the CTRP family may be an important biomarker of metabolic diseases in humans.

The biological function of CTRP7, a newly discovered member of the CTRP family, has been elusive since it was first identified (13). Previous animal studies found that CTRP7 mRNA expression in the fat of ob/ob diabetic mice was increased at eight-week-old (14). At the age of 12 weeks, blood glucose in ob/ob mice decreased due to a compensatory increase of insulin secretion. With decreased blood glucose, the expression of CTRP7 returned to the level of the control group (15). These findings suggest that CTRP7 may be involved in glucose metabolism *in vivo*. Another study reported that CTRP7 expression was upregulated in skeletal muscle in aged rats and down-regulated in caloric restricted animals. Therefore, it is further revealed that CTRP7 is related to energy metabolism (16). However, there are few studies on the relationship between CTRP7 and metabolic diseases in humans and animals, especially in MetS patients.

In this study, we examined serum CTRP7 and adiponectin (APN), an insulin sensitizer, levels in newly diagnosed MetS patients and healthy adults, and explored the association between CTRP7 and IR, APN, and metabolic parameters.

## Materials and Methods

### Study Populations

A cohort of 624 people participated in the study, including 310 males and 314 females aged 21-82 years. These individuals included 328 newly diagnosed patients with MetS, and 296 age-matched normal adults. MetS patients come from outpatients and inpatients in the Department of Endocrinology and Metabolism. MetS was diagnosed according to the criteria of the Chinese Diabetes Association (CDS guidelines 2017) (17). Individuals who meet three or more of the following conditions were considered for the diagnosis of MetS: 1) Central obesity, waist circumference (WC) ≥ 90 cm for men or ≥85 for women; 2) Triglyceride (TG) ≥ 1.7 mmol/L; 3) High-density lipoprotein-cholesterol (HDL-C) < 1.04 mmol/L); 4) Blood pressure (BP) ≥ 130/85 mmHg or receiving antihypertensive drugs; 5) Fasting blood glucose (FBG) ≥ 5.6 mmol/L or 2-hour blood glucose (2h-BG) ≥ 7.8 mmol/L or T2DM. Exclusion criteria include patients with liver cirrhosis, heart, liver, or renal failure, steroid use, various malignant tumors, infection, or other diseases. Individuals who adhered to daily exercise, smoking and alcohol dependence were also excluded from this study.

Healthy participants were selected through advertising, routine physical examination, or from the community. The diagnosis of impaired glucose tolerance (IGT) and T2DM is based on WHO standards in 1998 (18). In our cohort, MetS patients were newly diagnosed and did not use drugs or lifestyle interventions. The healthy controls had normal blood glucose, no family history of T2DM and hypertension, no clinical evidence of disease, and no medication. This study was approved by the Human Research Ethics Committee of Chongqing Medical University and was registered at chictr.org (ChiCTR2000032878). In the current study, subjects who met the inclusion criteria were randomly assigned numbers, and individuals with or without Mets were selected randomly according to the number to eliminate the selection bias. all subjects signed informed consent. The study was performed in accordance with the Helsinki Declaration.

### Anthropometric and Biochemical Measurements

Weight and height were measured using standardized equipment, and participants wore light indoor clothing without shoes. Body mass index (BMI) was calculated as weight divided by height squared (kg/m^2^). Waist circumference (WC) was measured by the lower border of the ribs and the iliac crest. The waist-to-hip ratio (WHR) was calculated by WC and hip circumstance (HC). Blood pressure was measured with a mercury sphygmomanometer. Right arm blood pressure was measured three times in resting and sitting posture. The second and third average readings of blood pressure were taken for calculating. Body adiposity index (BAI) was calculated as [HC (cm)/(height (m))^1.5^ − 18] (19). Visceral adiposity index (VAI) _Females_= WC/[36.58 + (1.89 × BMI)] × (TG/0.81) × (1.52/HDL) or Visceral adiposity index (VAI) _Males_= WC/[39.68 + (1.88× BMI)] × (TG/1.03) × (1.31/HDL) (20). After an overnight fasting, blood samples were collected, refrigerated, and serum samples were transported to the central laboratory for biochemical measurements within 12 h. Blood glucose and HbA1c were measured using a glucose oxidase method and HPLC, respectively. Insulin, free fatty acids (FFAs), and blood lipid were measured using an autoanalyzer (Hitachi 747; Hitachi, Tokyo, Japan) as described previously (21). Homeostasis model assessment of IR (HOMA-IR) was calculated using the following equations: HOMA-IR = fasting insulin (FIns, mU/L) × fasting blood glucose (FBG, mmol/L)/22.5 (22).

### Interventional Tests

#### Oral Glucose Tolerance Test

An OGTT was engaged in all populations, and blood for CTRP7, glucose, and insulin measurements were drawn at indicated times as reported previously (22).

#### Hyperinsulinemic-Euglycemic Clamps

HECs were engaged on 32 healthy subjects (15 male; age 25.1± 2.3 years; BMI: 22.5 ± 2.7kg/m^2^) as previously reported (23). During HEC, regular insulin (1 mU/kg/min) was infused for 2 h, and a variable infusion of 20% glucose was administered to maintain blood glucose at baseline concentration. M-values were determined as the glucose infusion rate (GIR) during the stable period of the HEC and were related to body weight. Blood samples for CTRP7 and insulin measurement were obtained at indicated times (0, 80, 100,110 and 120 min). Serum samples were stored at -80°C for further analysis.

#### Lipid Infusion Study

32 healthy subjects were given a lipid infusion (20% Intralipid, 1.5 ml/min) for 4-hour. Blood samples were collected at different time points as previously published (22).

### Cytokine Measurements

Circulating CTRP7 levels were determined with an ELISA kit (sk00396-09, Aviscerabio science Inc., MA. USA) according to the manufacturer’s protocol. The detection limit of serum CTRP7 levels was 5-320 μg/L, and the intra- and inter-assay coefficients of variation (CV) were less than 5% and 10%, respectively. The ELISA kit has high sensitivity, good specificity for human CTRP7 detection without obvious cross-reaction, and interference. Circulating APN levels were also measured with an ELISA Kit following the manufacturer’s protocol (Aviscera Bioscience, sk00010-02). Intra- and inter-assay CV were 8% and 10%, respectively.

### Bioinformatics Analysis

A protein-protein interaction (PPI) network of CTRP7 gene was established by using the Database Search tool (version 11.0). An interaction score of 0.4 was considered a cut-off criterion, and the PPI was visualized. The cluster Profiler package was used for Gene Ontology (GO) and Kyoto Encyclopedia of Genes and Genomes (KEGG) pathway analyses (24). REACTOME enrichment analysis was completed by a STRING database (25). The list of annotated terms was obtained by GO, KEGG, and REACTION analysis. *P*-value < 0.05 was considered a statistical significance in GO, KEGG terms, and REACTOME analysis.

### Statistical Analysis

All statistical analyses were performed using SPSS, version 20.0. All data were Mean ± SE or median of the interquartile range. The distribution of data was examined by Kolmogorov- Smirnov test. The differences between the two groups were compared by *t*-test or Mann-Whitney U test. The association between CTRP7 and APN as well as metabolic parameters was determined using correlation analysis. Multivariate regression analyses were performed to investigate the associations between variables. A Cochran-Armitage trend test was performed to analyze the tendency of serum CTRP7 levels associated with MetS. We used receiver operating characteristics (ROC) curve analysis to determine the cut-off point of CTRP7 for predicting MetS. In the OGTT, the area under the glucose curve (AUCg) was determined according to the trapezoidal rule. In statistical analyses, *p* < 0.05 was considered significant.

## Results

### Serum CTRP7 Concentration Is Higher in Individuals With MetS or IR

**Table 1** showed the main clinical and biochemical indicators in MetS patients and healthy controls. As expected, MetS patients have higher blood pressure (BP), obesity-related indicators (WC and BMI), glucose metabolism-related parameters [FBG, 2-h blood glucose after glucose overload (2h-BG), HbA1c and AUCg], triglyceride (TG), total cholesterol (TC), low-density lipoprotein cholesterol (LDL-C), FFA, FIns, 2-h serum insulin after glucose overload (2h-Ins), visceral adiposity index (VAI), and body adiposity index (BAI) and HOMA-IR than those of normal controls. However, high-density lipoprotein cholesterol (HDL-C) and APN levels were lower in MetS patients.

**Table 1:** Main clinical features and serum CTRP7 levels in MetS and control subjects.

Characteristics	Overall (n = 624)	MetS		*p*
		No (n = 296)	Yes (n = 328)	
Male	310	145	165	0.749
Age (yr)	53 (49-61)	52 (49-61)	54 (48-61)	0.217
WC (cm)	86.0 (80.0-91.0)	81.0 (77.0-86.0)	90.0 (85.0-94.0)	<0.001
BMI (Kg/m^2^)	24.2 (22.6-26.7)	23.3 (22.0 -24.6)	25.7 (23.7-27.6)	<0.001
SBP (mmHg)	130 (120-143)	123 (115-133)	136 (127-152)	<0.001
DBP (mmHg)	81 (75-90)	78 (70-84)	86 (80-93)	<0.001
FBG (mmol/L)	6.15 (5.34-7.11)	5.45 (5.02-6.23)	6.70 (6.01-7.46)	<0.001
2h-BG (mmol/L)	9.30 (7.22-12.69)	7.45 (6.26-11.22)	12.09 (8.78-13.27)	<0.001
FIns (mU/L)	9.57 (8.26-12.06)	8.80 (7.93-9.74)	11.09 (9.00-13.15)	<0.001
2h-Ins (mU/L)	50.9 (34.1-74.4)	45.3 (31.8-61.1)	56.0 (37.0-85.2)	<0.001
TG (mmol/L)	1.82 (1.40-2.24)	1.48 (1.16-1.83)	2.07 (1.79-2.44)	<0.001
TC (mmol/L)	5.02 (4.53-5.36)	4.83 (4.46-5.36)	5.09 (4.64-5.36)	0.001
HDL-C (mmol/L)	1.23 (1.15-1.44)	1.34 (1.18-1.53)	1.20 (1.12-1.32)	<0.001
LDL-C (mmol/L)	2.85 (2.52-3.12)	2.71 (2.25-3.03)	2.94 (2.68-3.15)	<0.001
FFA (µmol/L)	0.60 (0.49-0.70)	0.54 (0.43-0.68)	0.64 (0.55-0.72)	<0.001
HbA1c (%)	6.3 (5.6-8.3)	5.8 (5.3-6.9)	7.7 (6.0-8.5)	<0.001
HOMA-IR	2.55 (1.99-3.65)	2.10 (1.83-2.62)	3.31 (2.35-4.23)	<0.001
AUC_g_	21.1 (16.8-28.7)	17.3 (14.6-23.4)	26.6 (20.2-31.0)	<0.001
AUC_i_	85.4 (63.7-114.2)	84.3 (66.7-101.2)	87.0 (59.0-131.9)	0.080
VAI	2.27 (1.58-2.97)	1.67 (1.16-2.34)	2.67 (2.17-3.47)	<0.001
BAI	28.9 (26.3-31.8)	27.9 (25.6-30.6)	29.9 (27.2-32.6)	<0.001
APN (mg/L)	6.81 (4.14-12.35)	8.46 (5.16-14.13)	5.66 (3.22-9.81)	<0.001
CTRP7 (μg/L)	155.1 (119.0-197.5)	127.5 (102.1-160.5)	188.7 (139.7-224.3)	<0.001
CTRP7 (adjusted)^*^	——	137.4 ± 3.3	190.9 ± 3.2	<0.001

Values are given as mean ± SE or median (Interquartile Range). MetS, Metabolic Syndrome; BMI, body mass index; SBP, systolic blood pressure; DBP, diastolic blood pressure; FBG, fasting blood glucose; 2h-BG, 2-h blood glucose after glucose overload; FIns, fasting plasma insulin; 2h-Ins, 2-h serum insulin after glucose overload; TG, triglyceride; TC, total cholesterol; HDL-C, high-density lipoprotein cholesterol; LDL-C, low-density lipoprotein cholesterol; FFA, free fatty acid; HOMA-IR, homeostasis model assessment of insulin resistance; AUC_g_, the area under the curve of glucose during oral glucose tolerance test; AUCi, the area under the curve of glucose during insulin tolerance test; VAI, visceral adiposity index; BAI, body adiposity index; APN, adiponectin; ^*^Mean ± standard error by general linear model with adjustment of age and Sex.

**Figure 1:**
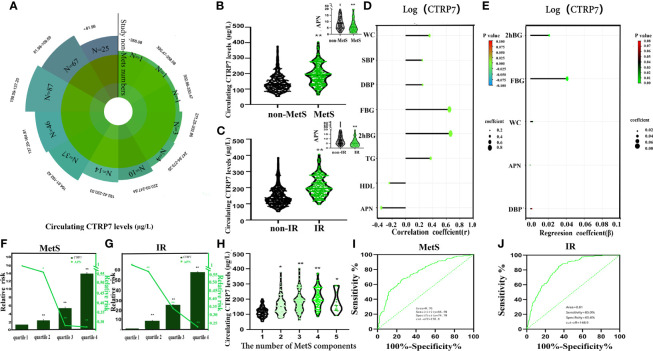
Circulating CTRP7 level and its relationship with metabolic markers and Mets in the study population. **(A)** Distribution of serum CTRP7 in the healthy population. **(B)** Serum CTRP7 and APN levels in MetS and healthy subjects. **(C)** Serum CTRP7 levels, according to HOMA-IR (IR, HOMA-IR > 3; non-IR, HOMA-IR ≤ 3). **(D)** Simple correlation analysis of variables associated with circulating CTRP7. **(E)** Multiple regression analysis of variables associated with circulating CTRP7. **(F, G)** The odds ratio of having MetS **(F)** and IR **(G)** in different quartiles of serum CTRP7 and APN. **(H)** Circulating CTRP7 levels to the number of MetS components. **(I, J)** ROC curve analyses for the prediction of MetS **(I)** and IR **(J)** according to serum CTRP7 levels. MetS, metabolic syndrome; IR, insulin resistance. Data are expressed as mean ± SE or median (Interquartile Range). *p < 0.05, **p < 0.01 *vs.* non-MetS or non-IR. quartile 1 or number 1.

As shown in **Figure 1A**, the distribution of circulating CTRP7 levels in healthy subjects ranged from 52.4 to 378.1μg/L, and 90% of the healthy population was between 76.0 μg/L to 231.6 μg/L. Importantly, circulating CTRP7 levels were significantly higher in MetS patients than those in control subjects. After adjusting the gender and age of the study populations, the levels of CTRP7 in patients with MetS were still markedly higher than those in the control group (**Figure 1B** and **Table 1**). In contrast, serum APN concentrations, an insulin sensor, were lower in MetS patients compare with those in controls (**Table 1** and **Figure 1B**). There were no difference in serum CTRP7 levels between males and females [159.0 (117.4-209.2) *vs.* 150.0 (119.1-192.8)μg/L].

To investigate the relationship between CTRP7 and IR, study populations were divided into IR and non-IR according to HOMA-IR > 3 or ≤ 3 (24). The results showed that IR population had higher serum CTRP7 levels [195.4 (163.3-244.7) *vs.*130.6 (103.1-173.6) μg/L; *p* < 0.01, **Figure 1C**] and lower APN levels [5.19 (2.90-8.76) *vs.* 8.07 (4.67-1.39) mg/L, *p* < 0.01) compared with non-IR population (**Figure 1C**).

### Relationship Between Serum CTRP7 and Other Indexes

Next, we engaged a linear correlation analysis. The results showed that CTRP7 was positively correlated with WC (r = 0.36,*p* < 0.01), SBP (r = 0.25,*p* < 0.01),DBP (r = 0.25, *p* < 0.01) FBG (r = 0.65,*p* < 0.01), 2h-BG (r = 0.66,*p* < 0.01), and TG (r = 0.37,*p* < 0.01), but negatively correlated with HDL-C (r = -0.24,*p* < 0.01) and APN (r = -0.36,*p* < 0.01; **Figure 1D**). In addition, multiple stepwise regression analysis uncovered that FBG, 2h-BG, WC, APN and DBP were independent impacted factors with serum CTRP7 concentration (**Figure 1E**). The multiple regression equation was: Y_log (CTRP7)_=1.422 + 0.021X_2h-BG_ + 0.041X_FBG_ + 0.003 X_WC_-0.000023X_APN_ + 0.001 X_DBP_.

### CTRP7 Is Related to the MetS and IR

Next, we engaged a multivariate logistic regression analysis to investigate the association of CTRP7 with MetS and IR. This analysis uncovered that circulating CTRP7 was markedly associated with MetS and IR, even if controlling for age and gender. However, BMI and lipids may contribute the most (**Table 2**). Using the row mean score difference and Cochran-Armitage test, we found that the increase of serum CTRP7 concentration showed a linear trend, and CTRP7 was independently related to MetS and IR (**Table S1**). In addition, we divided CTRP7 and APN concentration into four quartiles (quartile 1, <119.0µg/L; quartile 2, 119.0-155.0 µg/L; quartile 3,155.0-197.5µg/L; quartile 4, > 197.5µg/L for CTRP7 and tertile1, < 4.14mg/L; quartile 2, 4.14-6.81mg/L; quartile 3, 6.81-12.35 mg/L; quartile 4, >12.35 mg/L for APN). The relative risk of MetS and IR was calculated by logistic regression analysis. We found that the odds ratios for MetS was higher in quartiles 2, 3 and 4 of CTRP7 concentration than that of quartile 1 (95% CI 1.45-3.83 for quartile 2; 95% CI 3.32 - 8.88 for quartile 3 and 95% CI 8.15-24.33 for quartile 4 *vs.* quartile 1, all *p* < 0.01), while the odds ratio of MetS in the quartile 2, 3 and 4 of APN concentration was lower than that in quartile 1 (95% CI 0.34 - 0.88 for quartile 2; 95% CI 0.17 - 0.45 for quartile 3 and 95% CI 0.17- 0.44 for quartile 4; *vs.* quartile 1, *p* < 0.01 or 0.05) (**Figure 1F**). Additionally, the odds ratio of CTRP7 and APN concentration for predicting the development of IR was similar to that of MetS (95% CI 3.65 - 21.71 for quartile 2, 95% CI 10.42 - 59.92 for quartile 3, and 95% CI 23.93-140.45 for quartile 4 *vs*. quartile 1 for CTRP7; 95% CI 0.36-0.88 for quartile 2, 95% CI 0.21- 0.54 for quartile 3 and 95% CI 0.14-0.37 for quartile 4 *vs*. quartile 1 for APN, all *p* < 001) (**Figure 1G**). When circulating CTRP7 levels were stratified by MetS components including BP, blood lipids, abdominal obesity, and FBG, the circulating CTRP7 levels in patients with two or more MetS components were higher than those with one MetS component (**Figure 1H**). Patients with 2, 3, 4, or more MetS components had CTRP7 concentration for 118.2 (96.5-142.6), 150.1 (117.4-184.3), 180.6 (133.2 -207.6), 195.9 (163.8 -257.4) and 158.1(126.5-245.2) µg/L, respectively. We further used the ROC curves of circulating CTRP7 to predict the occurrence of MetS and IR. The area under the ROC curves for MetS (AUC_MetS_) and IR (AUC_IR_) was 0.76 with 66.5% sensitivity and 74.7% specificity for MetS (**Figure 1I**) and 0.81 with 85% sensitivity and 65.6% specificity for IR (**Figure 1J**), respectively. The optimal cut-off points of CTRP7 for MetS and IR were 158.8 μg/L and 148.9μg/L, respectively.

**Table 2:** Association of circulating CTRP7 with IR and MetS in fully adjusted models.

Model adjust	MetS			IR			
	OR	95% CI	*P*	OR		95% CI	*P*
Age	2.401	2.029-2.843	<0.001		3.194	2.621-3.893	<0.001
Age, Sex	2.410	2.035-2.855	<0.001		3.335	2.717-4.094	<0.001
Age, Sex, BP	2.206	1.841-2.643	<0.001		3.104	2.523-3.819	<0.001
Age, Sex, BP, BMI	1.880	1.549-2.282	<0.001		2.907	2.347-3.600	<0.001
Age, Sex, BP, BMI, WC	1.912	1.556-2.350	<0.001		2.875	2.319-3.564	<0.001
Age, Sex, BP, BMI, WC, Lipids	1.006	1.001-1.011	0.012		1.014	1.010-1.018	<0.001

Results of multivariate logistic regression analysis are presented as the odds ratio of being in IR and MetS status increase in serum CTRP7. CI, confidence interval; OR, odds ratio.

### Alternations of Serum CTRP7 Concentration in Different Intervention Studies

**Figure 2:**
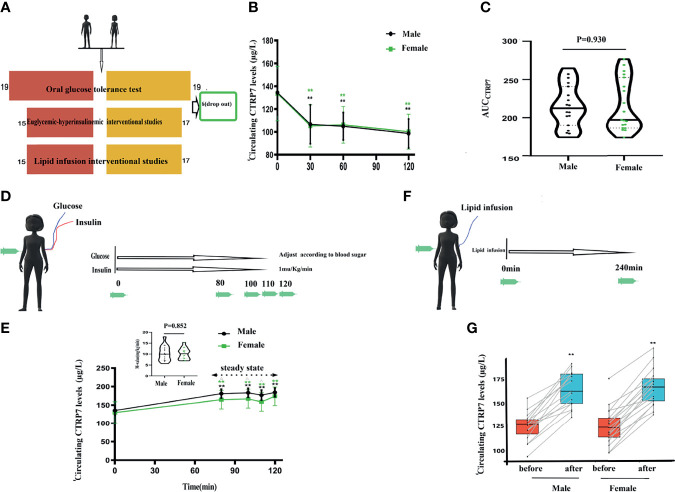
Circulating CTRP7 levels in the interventional studies of healthy individuals. **(A)** Schematic diagram of intervention study design. **(B)** Circulating CTRP7 concentrations during the OGTT. **(C)** Area under the curve of CTRP7 during the OGTT (AUC_CTRP7_). **(D)** Schematic diagram of the EHC. **(E)** Time course of serum CTRP7 alternations in healthy individuals during the EHC. **(F)** Schematic diagram of lipid infusion. **(G)** Time course of serum CTRP7 alternations in healthy subjects during lipid infusion. OGTT, oral glucose tolerance test; HEC, hyperinsulinemic-euglycemic clamp. Data are means ± SD. **p < 0.01 *vs.* baseline, or female.

The different intervention study designs are shown in **Figure 2A**. To evaluate whether serum CTRP7 is affected by blood glucose and insulin, we first conducted an OGTT study in normal men and women. In response to OGTT induced increases in blood glucose and insulin levels, the serum CTRP7 levels were significantly reduced in these subjects (**Figure 2B**). There was no significant difference in serum CTRP7 concentration between normal men and women (**Figures 2B, C**).

To further identify the regulatory role of blood glucose or insulin on circulating CTRP7, HECs were performed in 15 young men and 17 young women (**Figures 2D, E**). During the HEC, the blood glucose was clamped at the basal level (~ 5mmol), and insulin levels were significantly elevated from 48.3 ± 13.2 to 348.3 ± 52.4 pmol/L, indicating hyperinsulinemia *in vivo*. Our EHC results showed that exogenous increased insulin led to an obvious increase in circulating CTRP7 levels (from 135.6 ± 14.0 to 180.9 ± 12.2μg/L for men; 129.8 ± 29.2 to 164.7 ± 25.0 μg/L for women).

To further explore the relationship between CTRP7 and IR, we performed a lipid infusion to increase serum FFA levels and induce an acute IR *in vivo* (**Figures 2F, G**). The lipid infusion-induced increases in serum FFA levels and significantly increased the levels of circulating CTRP7 [from 127.8 (117.4-133.1) to 163.4 (150.5-181.4) μg/L for men; 119.3 (108.1-129.8) to 165.1(149.3-174.2) μg/L for women]. Therefore, we believe that FFA-induced IR promotes the release of CTRP7 *in vivo.*

### Bioinformatics Analysis

**Figure 3:**
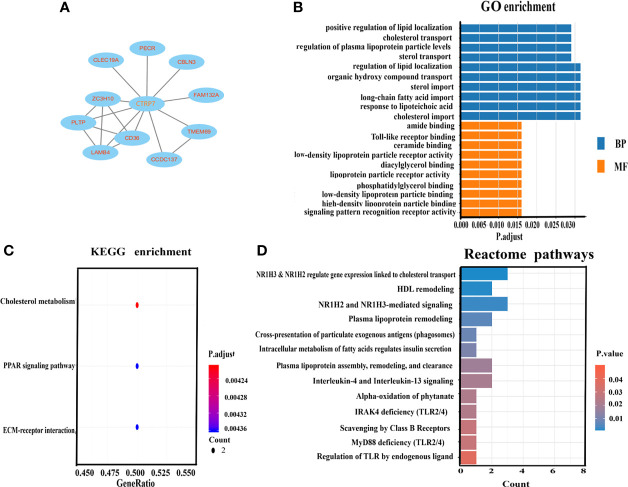
Bioinformatic analysis for CTRP7-related genes and signaling pathways. **(A)** Protein-protein interaction (PPI) network. **(B)** GO enrichment analysis for biological process (BP) and molecular function (MF). **(C)** KEGG enrichment analysis of the pathways. The gradual color represents the P-value. The size of the bubble represents the gene number. **(D)** REACTOME analysis for enriched pathways. The X-axis represents the number of involved genes. The Y-axis represents the pathway terms. GO, Gene Ontology; KEGG, Kyoto Encyclopedia of Genes and Genomes.

To further explore the relationship between CTRP7 and metabolic disorders, we performed bioinformatics analysis using Internet big data. As shown in **Figure 3A**, a PPI network was constructed. Ten genes (proteins) were involved in this PPI network, including ZC3H10, PECR, CBLN3, PLTP, CLEC19A, TMEM69, FAM132A, CCDC137, CD36, and LAMB4. Among them, some genes were related to lipid metabolisms, such as CD36 and FAM132A (26–28). For GO analysis, we used *p* < 0.05 as the screening condition and arranged the results from a large degree to a small degree. GO analysis revealed that in biological processes, the top 10 proteins include the positive regulation of lipid localization, cholesterol transport, regulation of plasma lipoprotein particle levels, sterol transport, regulation of lipid localization, organic hydroxy compound transport, sterol import, long-chain fatty acid import, response to lipoteichoic acid, cholesterol import. In the case of cellular components, no protein is enriched. Finally, in the case of molecular function, the top 10 proteins include the amide binding, Toll-like receptor binding, ceramide binding, low-density lipoprotein particle receptor activity, diacylglycerol binding, lipoprotein particle receptor activity, phosphatidylglycerol binding, low-density lipoprotein particle binding, high-density lipoprotein particle binding, signaling pattern recognition receptor activity [**Figure 3B**].

To explore the relationship between CTRP7 and signal pathway, we conducted a KEGG analysis. *p* < 0.05 was used as the screening condition, and the *p-*values were ranked from large to small. We found that CTRP7 related proteins were mainly enriched in cholesterol metabolism PPAR signaling pathway, ECM receptor interaction, etc. (**Figure 3C**). In addition, REACTOME enrichment analysis revealed that CTRP7-related genes were mainly enriched in metabolic-related signal pathways, such as cholesterol transport, HDL remodeling, lipoprotein remodeling, plasma lipoprotein clearance, and the relation of insulin secretion, etc. (**Figure 3D**).

## Discussion

In this study, we found that serum CTRP7 levels were significantly increased in MetS and IR individuals, and CTRP7 levels were associated with the disorder of glucose and lipid metabolism. Blood glucose, WC, APN, and BP were independent factors of circulating CTRP7. In addition, logistic regression analysis showed that high serum CTRP7 concentration was significantly associated with the occurrence of MetS and IR. We also found no significant difference in circulating CTRP7 levels between men and women, indicating that there may be no correlation between CTRP7 and sex hormones in study population. In the intervention studies, the OGTT test showed to a decrease in serum CTRP7 level, while the HEC test showed an increase in CTRP7 level. In addition, lipid infusion also increased serum CTRP7 levels. Bioinformatics analysis further showed that CTRP7 was associated with genes and signaling pathways related to glucose and lipid metabolism.

Cytokines, especially adipocytokines, and genetic and environmental factors play an important role in the pathogenesis of MetS (28, 29). It has been well documented that APN is an insulin sensitizer, and plays an important role in the pathogenesis of IR, diabetes, and metabolic disease (30–32). Low circulating APN levels are associated with MetS components, such as hyperglycemia, hyperinsulinemia, and dyslipidemia (33–36). In the current study, we find that increased serum CTRP7 levels are significantly associated with decreased circulating APN levels in MetS patients, as well as with other MetS components such as BP, blood glucose, WC and TG, etc. Logistic regression and ROC curve analysis reveals that circulating CTRP7 is significantly related to the occurrence of MetS.

Similar to our results, a small sample study in obese individuals (n = 37) showed that the level of serum CTRP7 in obese individuals was significantly increased and positively correlated with BMI, glucose, insulin, and HOMA-IR (11). In addition, one study reported decreased circulating CTRP7 levels in coronary artery disease (CAD) patients and may serve as a biomarker of CAD (37). Another study revealed that the production of CTRP7 in muscle tissue was increased and further increased by caloric restriction in old animals (16). These data further suggest that CTRP7 is a secretory protein related to metabolism. Given the previous study in obese individuals and our above results, including the reverse change in serum CTRP7 and APN concentration, and the negative correlation between CTRP7 and APN, we believe that CTRP7 has a negative regulatory effect on metabolism and insulin sensitivity. Therefore, it may be a metabolic inhibitor *in vivo.*

To further explore the factors regulating the secretion and release of CTRP7 *in vivo*, we conducted a variety of intervention studies. As a response to the oral glucose challenge, serum CTRP7 levels decreased significantly during the OGTT. Results from OGTT indicated a relevant link between CTRP7 secretion and glucose and insulin levels *in vivo.* Furthermore, HEC resulted in a significant increase in circulating CTRP7 levels when blood glucose was maintained at basal levels. We considered that increased insulin levels may promote the secretion or release of CTRP7 *in vivo*. Therefore, combined with the results of the OGTT and EHC, we believe that hyperglycemia inhibits the secretion and release of CTRP7. On the other hand, hyperinsulinemia stimulated its secretion and release. However, the inhibitory effect of blood glucose on CTRP7 may be stronger than the role of insulin on the secretion and release of CTRP7.

It is generally believed that the increase of FFA can lead to IR (36). To further explore whether elevated serum FFA has an effect on circulating CTRP7 levels, we performed a 4-hour intralipid infusion plus EHC in normal individuals. The results showed that the increased serum FFAs stimulated the secretion and release of CTRP7, resulting in a significant increase of circulating CTRP7 levels. This result also suggested that CTRP7 is related to acute-IR induced by elevated FFAs and maybe a potential nutrient sensor involved in lipid metabolism.

Finally, we used the network gene database and bioinformatics platform to evaluate the association of CTRP7 and other metabolism-related genes and signal pathways. Bioinformatics analysis showed that CTRP7 was related to lipid-metabolism genes, such as CD36 and FAM132A. KEGG analysis also showed that CTRP7 related proteins were mainly enriched in cholesterol metabolism PPAR signaling pathway and ECM receptor interaction. Therefore, our bioinformatics analysis further revealed that CTRP7 is a gene related to metabolism. This result supports our cohort and intervention studies. We thus consider that CTRP7 may be used as a biomarker of MetS and other metabolic diseases.

There are also some limitations in the current study, including 1) the population included in this study was limited to the Han people. Therefore, our results need to be confirmed in the population of different races; 2) although we strictly controlled the selection criteria for the study cohorts, we cannot completely exclude residual confounding factors; 3) our cross-sectional study did not reflect the changes of circulating CTRP7 levels in the development of MetS and the impact after treatment. Therefore, a long-term follow-up study is necessary; Although it is difficult to find a direct causal relationship with the results of a cross-sectional study, we believe that the data provided by this study provide an interesting avenue for further study of the association of CTRP7 with glucose and lipid metabolism.

In conclusion, our data show that MetS patients have high circulating CTRP7 levels, and lower APN levels when compared to controls. Circulating CTRP7 is associated with metabolism and MetS, and is regulated by glucose, insulin and FFA. The novelty of this study is that circulating CTRP7 levels in MetS patients were determined for the first time, and the relationship between CTRP7 and glucose and lipid metabolism as well as insulin sensitivity was evaluated by various intervention methods. Therefore, our data highlight the role of CTRP7 in MetS and its potential use as a predictive biomarker of MetS in the future.

## Data Availability Statement

The original contributions presented in the study are included in the article/**Supplementary Material**. Further inquiries can be directed to the corresponding author.

## Ethics Statement

This study was approved by the Human Research Ethics Committee of Chongqing Medical University. The patients/participants provided their written informed consent to participate in this study.

## Author Contributions

WH, QL, SG, DL, MT, and LL researched and analyzed data. BZ, CC, and HL reviewed and edited the manuscript. GY and MY wrote and edited the manuscript, and is the guarantor of this work and, as such, had full access to all of the data in the study and take responsibility for the integrity of the data and the accuracy of the data analysis. All authors contributed to the article and approved the submitted version.

## Funding

This work was supported by research grants from the National Natural Science Foundation of China (81300670) and from the Science and Technology Program of the Health Bureau of Chongqing (2019ZDXM039). Natural Science Foundation Project of Chongqing CSTC (cstc2020jcyj-msxmX0952).

## Conflict of Interest

The authors declare that the research was conducted in the absence of any commercial or financial relationships that could be construed as a potential conflict of interest.

## Publisher’s Note

All claims expressed in this article are solely those of the authors and do not necessarily represent those of their affiliated organizations, or those of the publisher, the editors and the reviewers. Any product that may be evaluated in this article, or claim that may be made by its manufacturer, is not guaranteed or endorsed by the publisher.

## Abbreviations

MetS, metabolic syndrome; APN, adiponectin; OGTT, oral glucose tolerance test; EHC, euglycemic-hyperinsulinemic clamps; FFA, free fatty acid; WC, waist circumference; BP, blood pressure; FBG, fasting blood glucose; 2h-BG, 2-hour post–glucose load blood glucose; TG, triglyceride; HDL-C, high-density lipoprotein cholesterol.
