# Supplementary material for: Circulating CTRP7 Is a Potential Predictor for Metabolic Syndrome
Source: Front Endocrinol (Lausanne). 2021 Nov 11;12:774309. doi: 10.3389/fendo.2021.774309 (PMC8636004; doi:10.3389/fendo.2021.774309)
Supplement: Supplementary file 1 [file Table_1.docx]

Supplementary Material

**Table S1** Row mean scores differ and Cochran-Armitage trend analysis of the impact of circulating CTRP7 level on IR and MetS

|  | MetS | | IR | |
| --- | --- | --- | --- | --- |
| Model adjusted | χ2 | *p*-value | χ2 | *p*-value |
| Row Mean Scores Test | 117.0948 | < 0.0001 | 163.6484 | < 0.0001 |
| Cochran-Armitage Trend Test | -10.8276 | < 0.0001 | -12.7951 | < 0.001 |

The circulating CTRP7 levels in all subjects were cut-off, and adjusted for age, BMI, WHR, SBP, DBP and lipid profile.
